# Infantile Diarrhœa

**Published:** 1888-02

**Authors:** 


### *INFANTILE DIARRHŒA.*

The diarrhœas of infantile life may be divided into two classes: those characterized by an excessive fluidity of the fæces and those distinguished by greenish discharges. The latter variety is the more common, and to this we will now give our attention:

Green diarrhœa was, until recently, considered to be the result of enteritis, resulting from and causing digestive troubles. The green color was thought by some to be derived from the biliary pigments, and by others from decomposition of the blood in the presence of sulphur compounds. To Damaschius, Clado, Hayem and Lesage belongs the merit of casting new light upon these obscure questions. From the researches of these authors, it has been determined that there are two varieties of green diarrhœa.

The *first* is caused by derangement of stomachic and intestinal digestion. There is marked acidity of the stools, and a microscopic examination of the discharges reveals no special microbe, nor will the cultivation of any of the varieties found develop a green color.

The *second* variety is due to the presence of a special microbe, the *bacillus chromogenus.* The reaction of the stools is neutral. Thus we have the means of a rapid and accurate differential diagnosis.

While the former variety is not, as a rule, a serious disease, the latter will lead rapidly to a fatal termination, unless speedily combatted. The green diarrhœa, not caused by bacilli, is due to errors in diet. Blot and Gueniot have cited cases produced by over-feeding. The treatment consists principally in regulating the diet, and administering alkaline waters, such as Vichy or Aqua Calcis in ʒiii at each feeding.

The latter variety of green diarrhœa is liable to follow the former, or may be suddenly developed in previously healthy children. It differs from the former in being distinctly infectious, so that in an asylum a single case will soon develop a true epidemic. Air is the common vehicle of the microbe, although it may be carried in water or milk.

This form of diarrhœa requires a treatment almost contrary to the former; alkalies would aggravate the conditions, and acids are indicated. Hayem recommends a two per cent. solution of lactic acid given in drachm doses every hour or two, according to the severity of the case. The acid should not be given for a-half hour before or after nursing.—*Hayem and Lesage in Bulletin Medical.*

